# The Role of Methods for Applying Cucurbit[6]uril to Hydroxyapatite for the Morphological Tuning of Its Surface in the Process of Obtaining Composite Materials

**DOI:** 10.3390/ma17204995

**Published:** 2024-10-12

**Authors:** Tolkynay Burkhanbayeva, Arthur Ukhov, Dina Assylbekova, Zukhra Mussina, Irina Kurzina, Sandugash Abilkasova, Abdigali Bakibaev, Manar Issabayeva, Rakhmetulla Yerkassov, Zhanat Shaikhova

**Affiliations:** 1 Chemistry Department, Faculty of Natural Sciences, L. N. Gumilyov Eurasian National University, Astana 010000, Kazakhstan; erkass@mail.ru; 2 Chemistry Department, National Research Tomsk State University, Arkady Ivanov St. 49, 634028 Tomsk, Russia; artyryxov1@gmail.com (A.U.); kurzina99@mail.ru (I.K.); bakibaev@mail.ru (A.B.); 3 High School of Chemical Engineering and Biotechnology, M. Auezov South Kazakhstan University, Shymkent 160012, Kazakhstan; asylbekova.dina@inbox.ru; 4 Department of Chemistry, Chemical Technology and Ecology, Almaty Technological University, Almaty 050012, Kazakhstan; muszuhra07@gmail.com (Z.M.); sandugash.abilkasovaa@gmail.com (S.A.); zh.shaikhova1965@gmail.com (Z.S.); 5 Department of Chemistry and Chemical Technology, “Toraighyrov University” NCJSC, Pavlodar 140008, Kazakhstan; isabaeva.manar@mail.ru

**Keywords:** cucurbit[n]urils, hydroxyapatite, functional materials, composite materials

## Abstract

In this work, composite materials were obtained for the first time using various methods and the dependences of the resulting surface morphologies were investigated. This involves modifying the surface with cucurbit[n]urils, which are highly promising macrocyclic compounds. The process includes applying cucurbit[6]uril to the hydroxyapatite surface in water using different modification techniques. The first method involved precipitating a dispersion of **CB[6]** in undissolved form in water. The second method involved using fully dissolved **CB[6]** in deionized water, after which the composite materials were dried to constant weight. The third method involved several steps: first, **CB[6]** was dissolved in deionized water, then, upon heating, a dispersion of **CB[6]** was formed on the surface of **HA**. The fourth method involved using ultrasonic treatment. All four methods yielded materials with different surface morphologies, which were studied and characterized using techniques such as infrared (IR) spectroscopy and scanning electron microscopy (SEM). Based on these results, it is possible to vary the properties and surface morphology of the obtained materials. Depending on the method of applying **CB[6]** to the surface and inside the **HA** scaffold, it is possible to adjust the composition and structure of the target composite materials. The methods for applying **CB[6]** to the hydroxyapatite surface enhance its versatility and compatibility with the body’s environment, which is crucial for developing new functional composite materials. This includes leveraging supramolecular systems based on the **CB[n]** family. The obtained results can be used to model the processes of obtaining biocomposite materials, as well as to predict the properties of future materials with biological activity.

## 1. Introduction

In today’s era of personalized medicine, there is a pressing demand to develop new implant materials that possess specific attributes. For these materials to be effectively used in medical applications, they must exhibit an ideal blend of various properties.

Any bone grafts must have the following properties: be completely biocompatible, porous, serve as a matrix on the surface of which the recipient’s cells are fixed (osteoconductivity), gradually resorb and be replaced by newly formed bone (creeping substitution). For successful bone formation, it is also necessary to comply with two important requirements—good vascularization and mechanical stability of the implantation area. Bone grafts are divided into auto-, allo-, xenografts, synthetic and composite materials. In addition, their main characteristics of composite biomaterials include their composition, shape, structure, surface texture and mechanical properties. The decisive factor is their biocompatibility, i.e., they must interact with surrounding tissues without causing adverse reactions or inflammation. Additionally, it is advantageous if these materials encourage the growth of blood vessels or bone around them, aiding in the healing and repair of damaged tissue.

For implants or wound healing materials, these properties collectively influence how well the material interacts with surrounding tissues. Bioactive materials, in particular, can promote the growth of new tissues and expedite the healing process. Therefore, advancing these materials is crucial for enhancing the quality of medical care and improving treatment outcomes [1]. The effectiveness of personalized medicine heavily relies on advancements in developing new implant materials that possess all the essential characteristics for safe and efficient interaction with biological tissues. Innovations in this field are paving the way for better treatments for a range of diseases and injuries, offering patients quicker recovery and an enhanced quality of life.

Materials interacting with the internal environment of the body should be as non-toxic as possible for cells and tissues. However, many substances with pronounced antibacterial properties often turn out to be quite toxic, which leads to low biocompatibility. Biocompatibility, which is determined by the nature and degree of interaction of biomaterials with body tissues, is one of the key problems in biomaterial research [2,3,4]. This covers such processes as immune rejection or inflammatory tissue response to the presence of a foreign body. In this context, all biomedical devices must meet extremely stringent clinical requirements. Before being implanted into the human body, materials undergo meticulous surface modification to enhance their biocompatibility. Developing materials that are both biocompatible and possess specific biological activities is a challenging endeavor.

Furthermore, current clinical treatments often depend on oral or intravenous drug administration. This method results in high drug levels in the blood immediately after dosing, but the concentration rapidly drops below the therapeutic window. This fluctuation can lead to drug concentrations initially reaching toxic levels and then falling below therapeutic levels, reducing the effectiveness of the therapy.

Furthermore, current clinical treatments often depend on oral or intravenous drug administration. This method results in high drug levels in the blood immediately after dosing, but the concentration rapidly drops below the therapeutic window. This fluctuation can lead to drug concentrations initially reaching toxic levels and then falling below therapeutic levels, reducing the effectiveness of the therapy [5].

Drug-releasing implants have emerged as a promising alternative to traditional oral and intravenous drug administration for various clinical applications. Materials currently used for developing implants with controlled drug release include titanium nanotubes, porous silicon, polymers, and various microtechnologies [6].

Drug-releasing implants offer several unique advantages. They can deliver sustained, remotely controlled, programmable, and localized drug release directly to the target site, enhancing therapy effectiveness and minimizing side effects for patients. These capabilities are challenging to achieve with conventional systemic drug administration [6].

Bioactive materials occupy a special place among implants, as they are able to stimulate the growth of new tissue around them. This ability can significantly improve the healing and recovery processes, which is especially important in medical applications related to the treatment of wounds and injuries. Bioactive materials accelerate tissue regeneration, which makes them indispensable in medical practice.

All characteristics of implant materials that make them biosimilar are crucial for their medical applications. Research and development in this field are continuously advancing, aiming to discover the best materials that meet all requirements and deliver optimal results for patients. Innovations in the development of these materials could lead to significant improvements in treating various diseases and injuries, offering patients faster and more effective recovery.

Thus, the creation and use of drug-releasing implants open new horizons in medical science and practice. These implants not only enhance the effectiveness of therapy but also significantly improve patients’ quality of life by minimizing side effects and providing more precise and targeted treatment [7].

One of the potentially promising materials, in this context, is modified hydroxyapatite (**HA**) enriched with biologically active compounds. Hydroxyapatite is a key component of bones, comprising approximately 50% of their total mass, and teeth, where its content reaches 96% in enamel. Additionally, **HA** is the most suitable ceramic material for artificial teeth or bones due to its excellent biocompatibility and bioactivity [8]. This can be either synthetic or natural. In medicine, synthetic **HA** is used as a filler to repair lost bone tissue and as a coating on implants to promote new bone growth [9]. Pure **HA** is not found in biological systems, but due to its chemical similarity to bone and tooth minerals, it is commonly used as a coating on orthopedic implants (e.g., hip replacements) and dental implants [8,10,11,12,13,14]. **HA** bioceramics are also highly popular [15,16]. Due to its close similarity to biological apatite, **HA** has long been utilized in liquid chromatography for nucleic acids, proteins, and other biological compounds [17,18], as well as for drug delivery purposes [19]. Additionally, **HA** is included in some toothpaste brands as a mild polishing agent, replacing calcium carbonate [20]. These materials are inert to living tissues and exhibit low toxicity, resulting in a minimal risk of allergic reactions, inflammation, and mutagenic effects. Moreover, modified hydroxyapatites can speed up reparative osteogenesis at the implantation site and enhance the proliferation of osteoblasts [21].

Methods for modifying the surface of porous materials by saturating them with biologically active compounds, including macrocyclic compounds, are becoming increasingly relevant. These methods provide the opportunity to control the release of antibiotics, drugs, biologically active substances and cells. Modified hydroxyapatites have the potential to significantly improve the processes of healing and tissue regeneration, which makes them valuable in medicine [22].

Thus, advancing materials with enhanced biocompatibility and bioactive properties is a crucial challenge in biomaterials science. Ongoing research and development in this area, including innovative approaches to modifying hydroxyapatite and other materials, offer new opportunities to enhance treatment effectiveness and improve patient quality of life [22].

Macrocyclic compounds are often preferred over other drug delivery systems such as dendrimers, liposomes, micelles, carbon nanotubes, hydrogels, and polymers due to their advantages, including increased stability and more controlled drug release rates. [23,24,25].

Additionally, macrocyclic compounds exhibit high resistance to external factors, enabling them to retain their properties over extended periods. They offer a more predictable and sustained release of active substances, which reduces dosing frequency and enhances therapy effectiveness. The controlled release rate helps maintain optimal drug concentrations in the body, minimizing the risk of side effects and improving treatment outcomes [23,24,25].

These effective delivery systems for drugs and biologically active substances represent a significant advancement in the development of new approaches in medicine and other scientific and technological fields. They have the potential to greatly enhance patients’ quality of life by ensuring more accurate and safe medication administration. Innovations in this area are also paving the way for new treatment possibilities for various diseases, including chronic and hard-to-treat conditions.

Research and development in macrocyclic delivery systems are advancing rapidly, promising even more sophisticated and effective solutions in the future. These advancements have the potential to drive significant breakthroughs in medicine, pharmaceuticals, and related fields, resulting in safer, more effective, and personalized treatments.

For effective modification of the surface of porous materials, suitable starting reagents include nitrogen-containing macrocyclic systems based on glycoluril and its derivatives, such as cucurbit[n]urils and bamboo[n]urils.

Previously, we developed and studied new materials based on the cucurbit[6,7,8]uril family (**CB[6,7,8]**) and hydroxyapatite (**HA**) as a carrier [26]. Our research revealed that these biocomposite materials demonstrated high biocompatibility and low toxicity in vitro [26]. We observed a similar effect when applying bambus[6]uril to the surface of titanium nickelide [27] and **HA** [28].

Thus, the aim of this work is to study various methods for obtaining composite materials based on **CB[6]** and **HA** in order to study and optimize the surface of the composites, as well as to select the optimal conditions for obtaining and potential application of these composites.

## 2. Materials and Methods

### 2.1. Instruments for Interpreting Results

#### 2.1.1. Infrared Spectroscopy

The identification and structure study of the obtained samples were carried out by FTIR spectroscopy on a Nicolet 6700 IR spectrometer from Thermo Fisher Scientific (Waltham, MA, USA). The samples were studied by the attenuated total internal reflection method in the spectral range from 400 to 4000 cm^−1^ with a resolution of 4 cm^−1^. The obtained reflection spectra were converted to absorption spectra using the Kubelka-Munk transformation.

#### 2.1.2. Scanning Electron Microscope (SEM) of **CB[6] + HA** Samples

System with electron and focused beams QUANTA 200 3D (FEI, Hillsboro, OR, USA), accelerating voltage: 200–30,000 V continuous, resolution: 3.5 nm at 30 kV in the ESEM mode, <15 nm at 3 kV in the low vacuum mode.

#### 2.1.3. X-ray Diffraction Analysis

The study of **CB[6]** crystalline powders was carried out using X-ray phase analysis. The samples were studied using a device—X-ray diffractometer XRD-7000 (Shimatdzu, Kyoto, Japan), anode: Cu, radiation wavelength Kα(Cu) = 1.5406 Å, measurement range 5–50° in 2θ, measurement speed 30°/min. Identification of the analyzed sample was carried out when the spectrum coincided with the diffraction pattern of the reference substances using diffraction data from The Cambridge Crystallographic Data Center.

### 2.2. Preparation of Scaffolds from HA

The synthesis of hydroxyapatite (**HA**) was carried out by the liquid-phase method using microwave radiation at pH ~ 11 according to the scheme [29]:

10Ca(NO_3_)_2_ + 6(NH_4_)_2_HPO_4_ + 8NH_4_OH → Ca_10_(PO_4_)_6_(OH)_2_ + 20NH_4_NO_3_ + 6H_2_O

To prepare the initial solutions, we used: calcium nitrate tetrahydrate Ca(NO_3_)_2_·4H_2_O, ammonium phosphate (NH_4_)_2_HPO_4_, 25% aqueous ammonia solution and distilled water.

A suspension of 47.20 g Ca(NO_3_)_2_·4H_2_O in 200 mL distilled water was dissolved in a 300 mL beaker to obtain a solution with a concentration of 0.5 mol/L. Similarly, 15.84 g (NH_4_)_2_HPO_4_ were dissolved in 200 mL distilled water to obtain a solution with a concentration of 0.3 mol/L. These solutions were mixed in a 500 mL beaker and the pH was adjusted to 11 by adding 25% aqueous ammonia with constant stirring. The beaker with the reaction mixture was covered with film and placed in a microwave oven, where the power was set to 100–150 W, after which the heating was turned on. Irradiation in the microwave was carried out until the mixture boiled. The contents of the reaction vessel were stirred every 15 min to avoid local overheating. The total heating time was 45 min. After that, the beaker with the contents was removed and left at room temperature for 48 h to form the hydroxyapatite phase. The precipitate was filtered, dried in an oven at 110 °C to constant weight (20 h), and then ground in a mill to a homogeneous state. From the obtained hydroxyapatite powder, carriers were formed in the form of tablets with a diameter of 2 cm and a thickness of about 1 mm, which were calcined at a temperature of 600 °C to constant weight and sintering of the material. According to the standard technique, the physicochemical parameters of the obtained compound are practically the same as in the literature: particle size 0.5–1 nm, specific surface area 106.0 m^2^/g, total pore volume 0.50 cm^3^/g, average pore size 20 nm [29].

**Figure 1:**
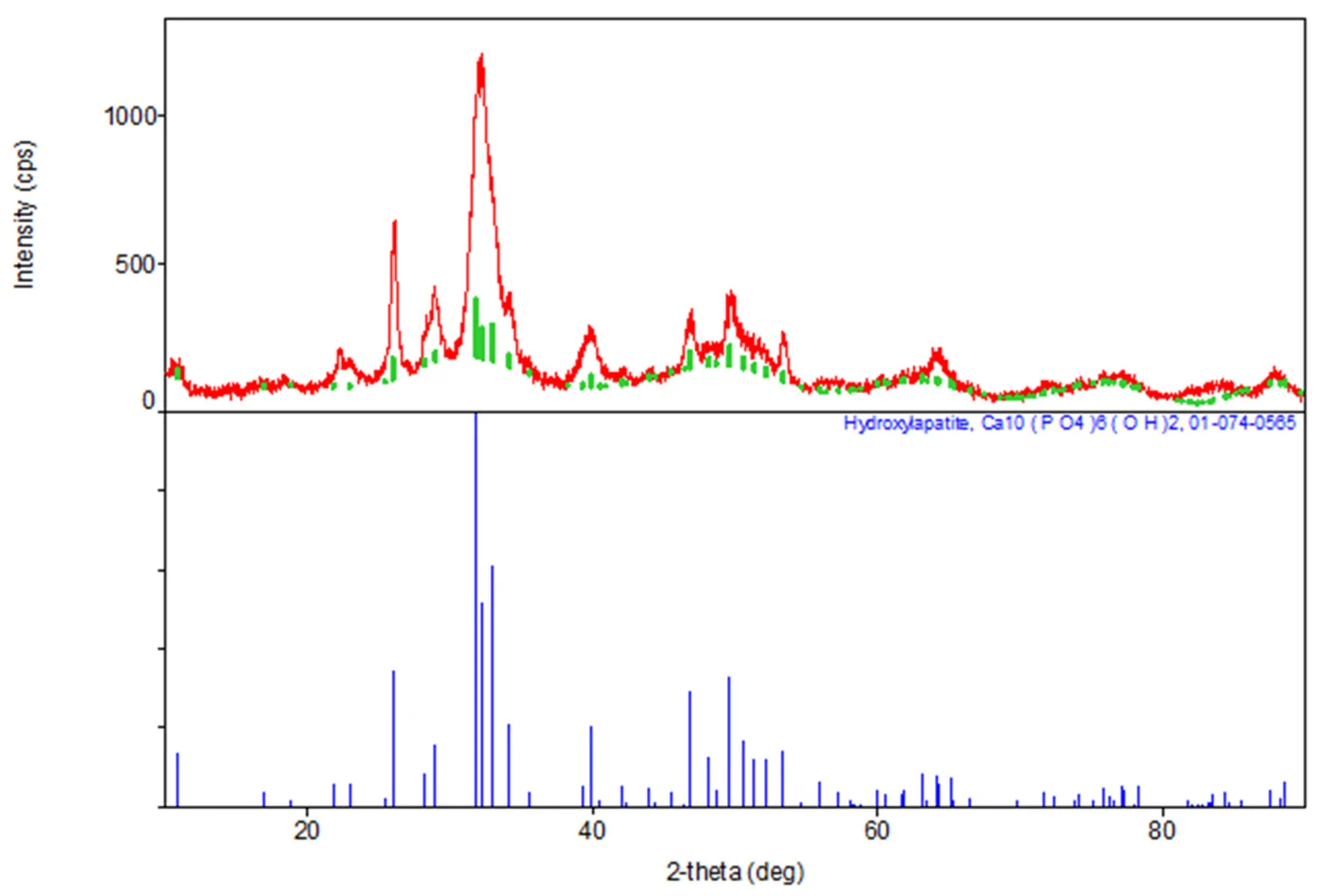
Diffractogram of synthesized stoichiometric **HA** (red line—synthesized HA, green line—standard).

The XRD results indicate the formation of a hexagonal system during the synthesis of stoichiometric **HA**, sp. gr. P63\m, the gross formula of which can be described by the formula Ca_10_(PO_4_)_6_(OH)_2_ (Table 1, Figure 1). The unit cell parameters of the synthesized **HA** are close in values to the tabulated data (JCPDS data, No. 9-432 [29]).

### 2.3. Synthesis and Purification of CB[6]

**Figure 2:**
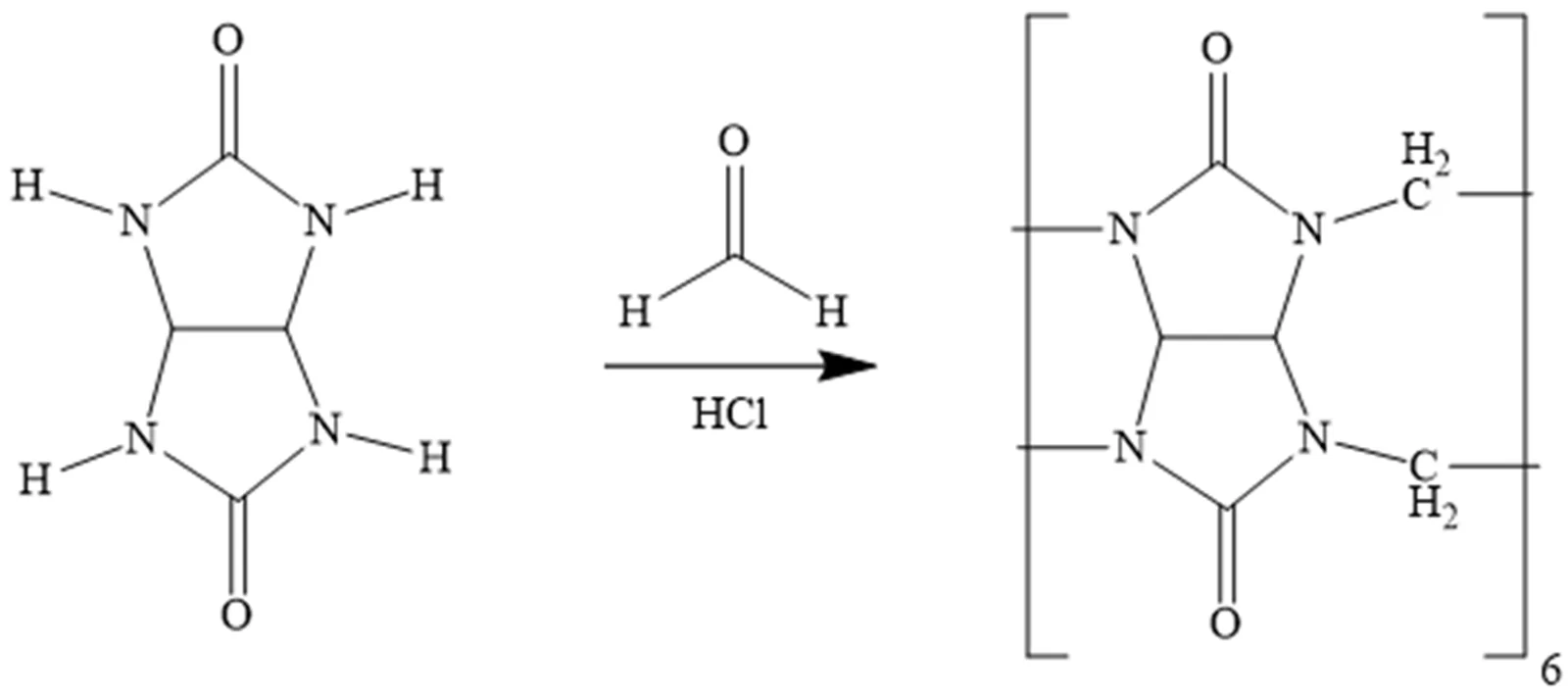
Synthesis scheme **CB[6]**.

Synthesis of **CB[6]** was carried out according to the procedure (Figure 2) [30]. 4.22 g (0.14 mol) of paraformaldehyde and 14 mL of 10 M sulfuric acid were placed in a three-necked flask with a magnetic stirrer and reflux condenser, after which the mixture was stirred until the paraformaldehyde was completely dissolved. Then, 10 g (0.07 mol) of glycoluril were gradually added to the flask in small portions to avoid premature oligomerization. The reaction mixture was maintained for 24 h at 95 °C.

**Figure 3:**
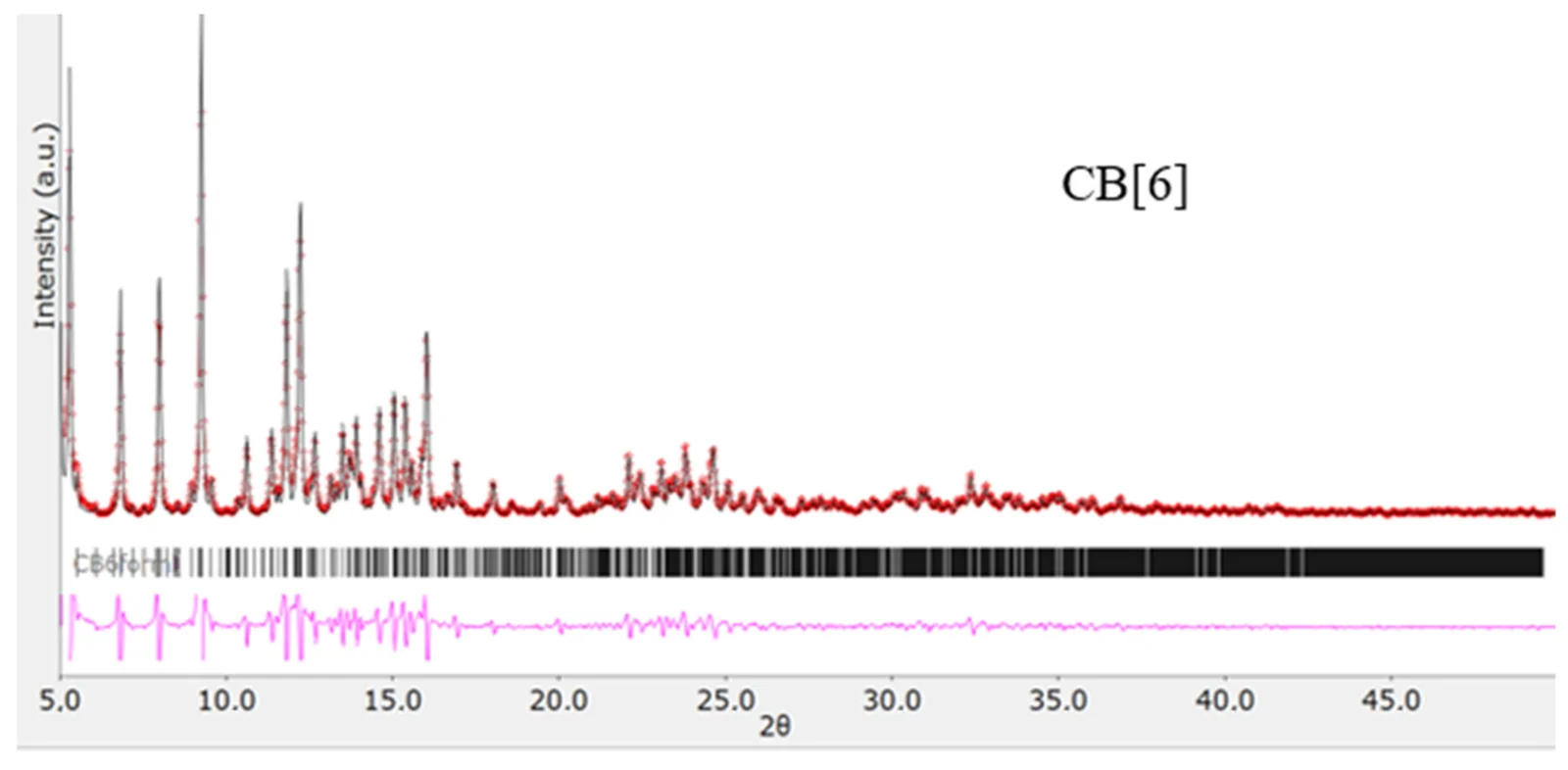
X-ray diffraction pattern **CB[6]**. Reprinted from Ref. [26].

After the reaction, the resulting precipitate was filtered off and recrystallized from an HCl solution (37%) to remove homologues and by-products of the reaction. Next, the crystalline **CB[6]** powder was kept in a vacuum oven at a temperature of 70 °C for 48 h to remove water of crystallization and hydrochloric acid. The structure of the resulting **CB[6]** was proven using X-ray phase analysis by comparing the diffraction patterns we obtained with the diffraction patterns available in the database of The Cambridge Crystallographic Data Center: **CB[6]** No. 7209204 (Figure 3).

## 3. Results and Discussion

**Figure 4:**
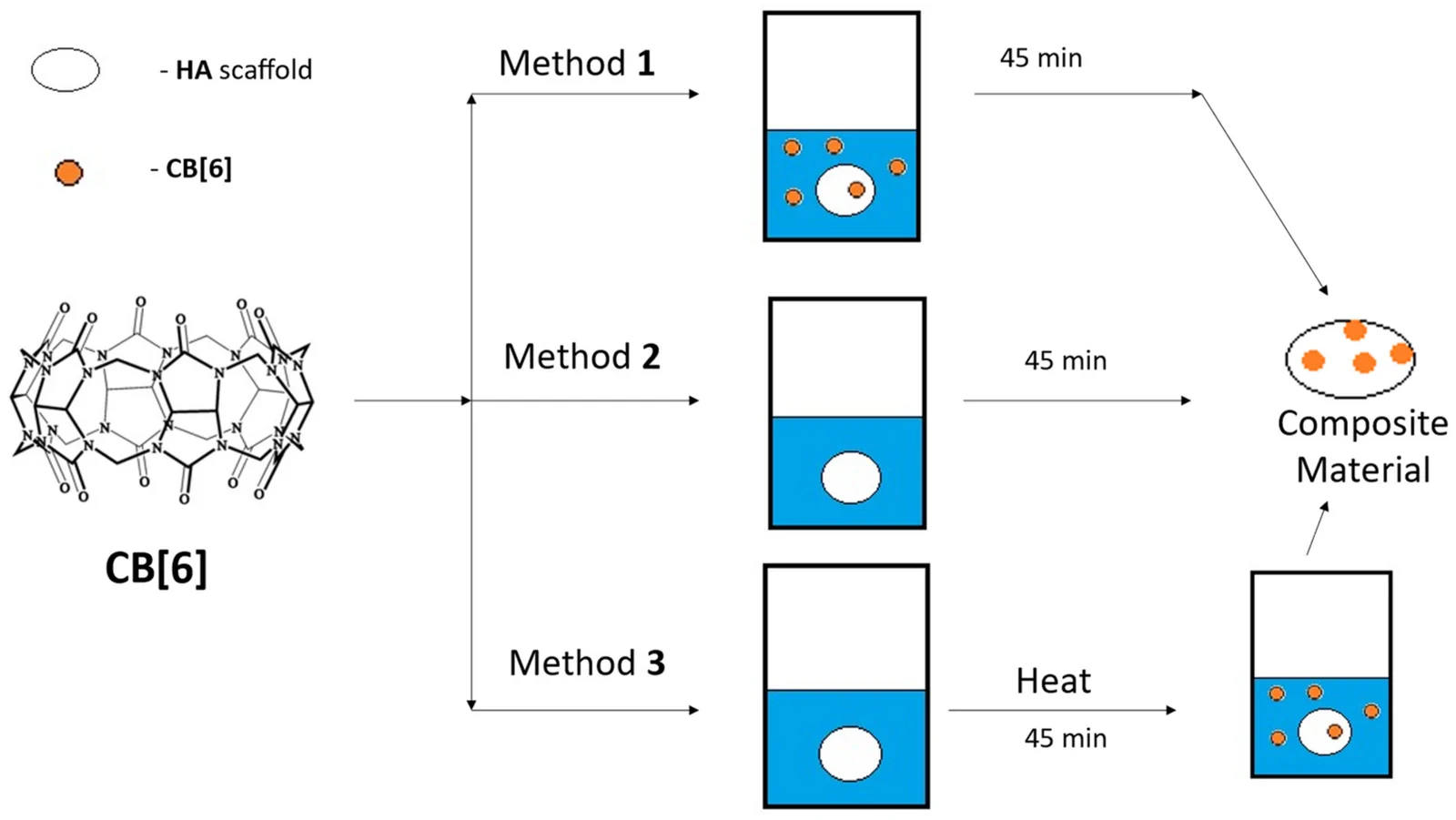
Scheme for applying **CB[6]** to the **HA** surface.

As part of this study, we have developed three methodological approaches to the features of obtaining materials based on **CB[6]** and **HA**, which differ in the method of applying the macrocycle to the scaffold surface (Figure 4).

In the first case (**Method 1**), **CB[6]** was applied from a dispersed solution. In this case, uncalcined **CB[6]**, which usually contains water inside the cavity [31,32]. Traditionally, recrystallization of **CB[6]** is carried out from an aqueous solution of hydrochloric acid [33], due to which its solubility in water is greatly reduced and a dispersion of the original macrocycle is formed. To implement **Method 1**, 15 mg of uncalcined **CB[6]** was placed in deionized water and stirred for 10 min until a dispersion was obtained. Next, an **HA** scaffold in the form of a **HA** tablet with a diameter of 2 cm and a thickness of 2 mm was placed into the resulting dispersed solution and kept for 45 min. Finally, the resulting composite was dried to constant weight (Figure 4).

Another method (**Method 2**) was that **CB[6]** was applied after calcining it at 60 °C for two days (48 h), which in our opinion increases the solubility of **CB[6]** in water. To implement **Method 2**, 15 mg of calcined **CB[6]** was dissolved in 15 mL of deionized water, after which the **HA** scaffold was immersed in the resulting solution for 45 min. Then, the resulting composite was dried at room temperature to constant weight (Figure 4).

The next method (**Method 3**) is that **CB[6]** was precipitated from solution—15 mg of calcined **CB[6]** was dissolved in 15 mL of deionized water. Next, the **HA** scaffolds were immersed in a **CB[6]** solution and heated at a temperature of 50–60 °C. Within 12 min, the solution became cloudy, which is obviously due to the fact that **CB[6]** molecules adsorbed water when heated and, as a result, the formation of a dispersion was observed. After 45 min, the composite was removed and dried at room temperature to constant weight (Figure 4).

**Figure 5:**
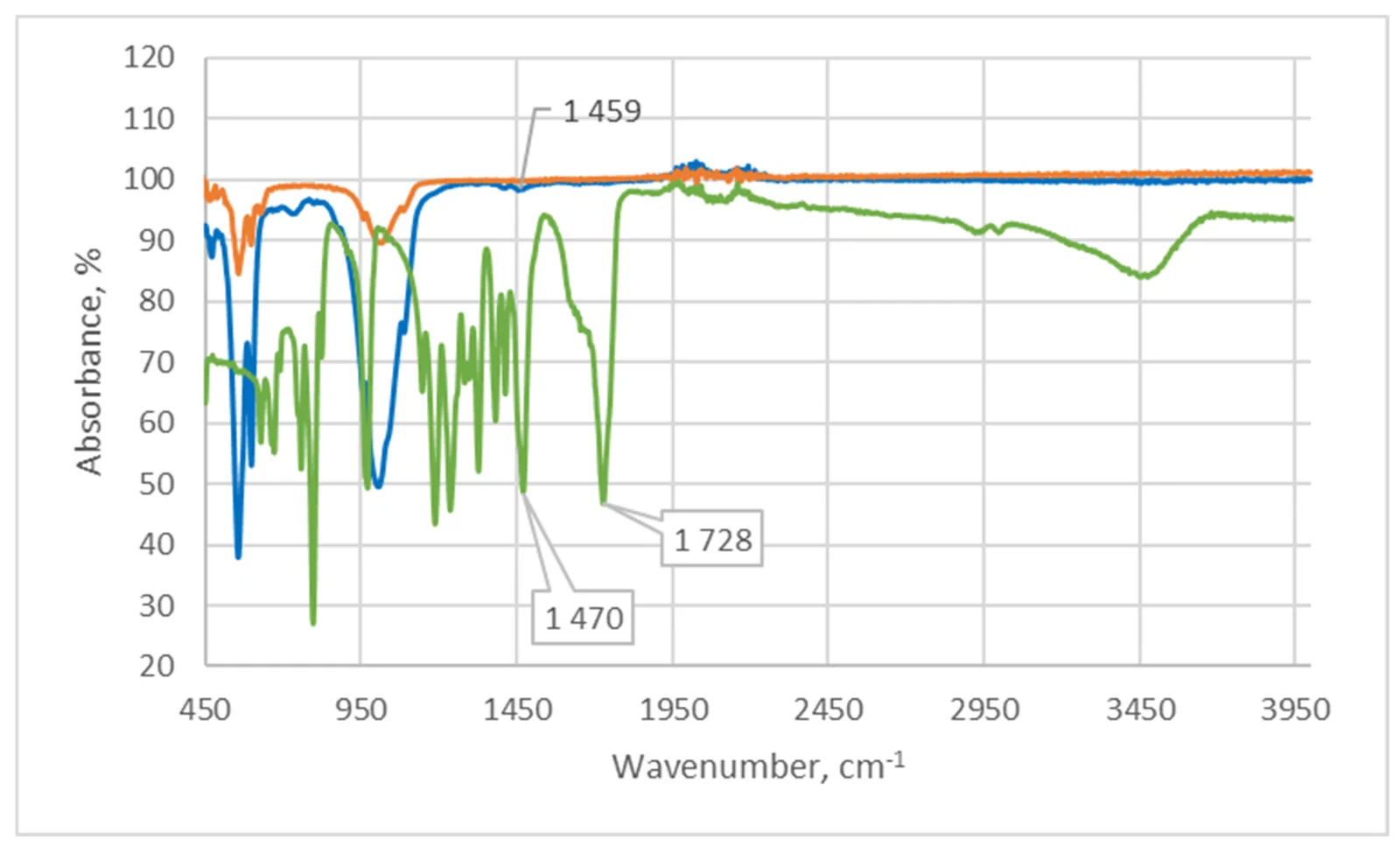
IR spectrum of the cleavage composite obtained by **Method 1**, **CB[6]** (green line), **HA** (orange line) and composite (blue line).

**Figure 6:**
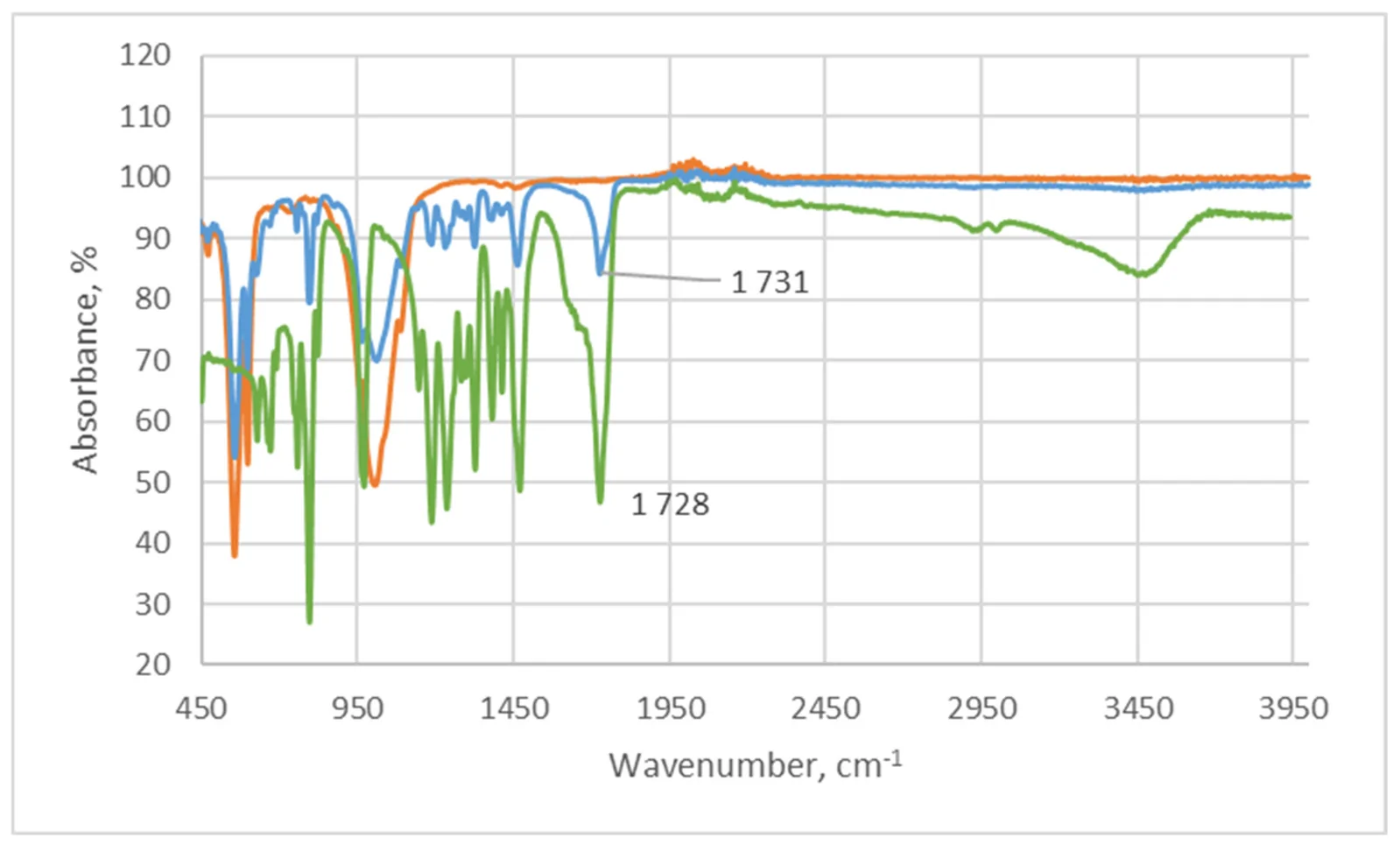
IR spectrum of the cleavage composite obtained by **Method 2**, **CB[6]** (green line), **HA** (orange line) and composite (blue line).

The composites obtained by **Methods 1–3** were studied using IR and SEM spectroscopy. Analysis of the IR spectra of samples obtained by **Methods 1–3** showed the following results. When the **HA** scaffold is immersed in a **CB[6]** dispersion (**Method 1**), the IR spectrum of the composite cleavage shows low intensity absorption bands associated with **CB[6]** (Figure 5). This fact suggests that most of the **CB[6]** is located on the surface of the scaffold, as convincingly evidenced by the SEM results (Figure 6).

When producing composites using **Method 2** (immersion in a **CB[6]** solution), the spectral picture is noticeably different. Thus, in the IR spectrum of the cleavage sample **CB[6] + HA**, clear absorption bands are observed, which belong to **CB[6]**. Thus, at 1731 cm^−1^ an absorption band is observed, which corresponds to the stretching vibration of the carbonyl groups (C=O) of the glycoluryl unit **CB[6]**. When compared with the IR spectrum of **CB[6]**, it is clear that there is a shift of 3 cm^−1^ to the long wavelength region, which indicates an insignificant interaction of the **HA** surface with **CB[6]**. At 1200–1500 cm^−1^, bending vibrations of CH_2_ groups are identified. When comparing the IR spectra of the **CB[6] + HA** composite with the IR spectrum of **CB[6]**, it is clear that the main absorption bands related to **CB[6]** are present in the spectrum, but there are no absorption bands at 3400 cm^−1^ (stretching vibrations -OH water groups) and 2900–3000 cm^−1^ (stretching vibrations of -CH-CH- groups), which is obviously due to the fact that **CB[6]** is in a lower concentration compared to HA and the signals have low intensity.

The sample obtained by **Method 3** shows a similar picture—absorption bands related to **CB[6]** are also identified inside the sample cleavage.

**Figure 7:**
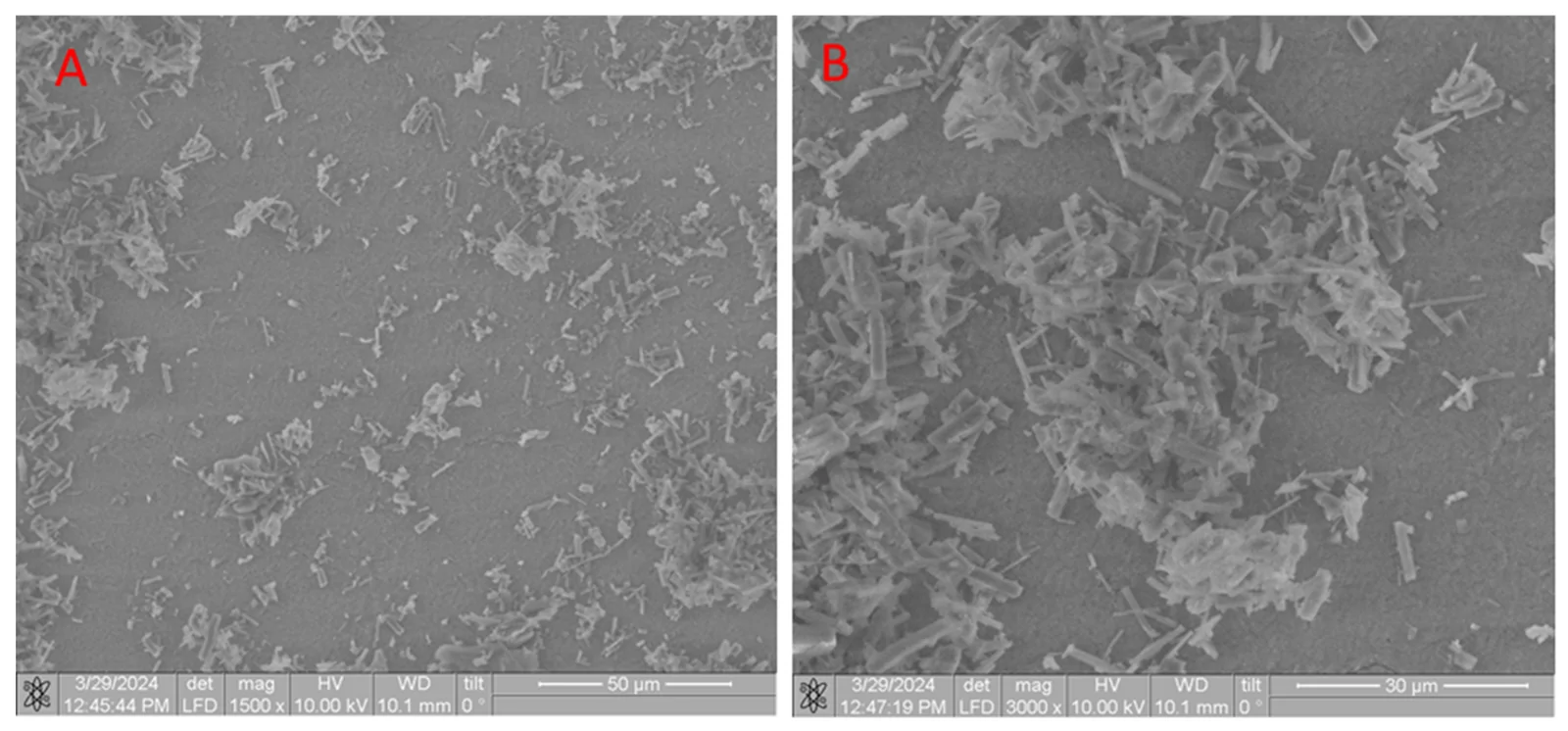
SEM images of samples obtained by **Method 1** ((**A**) 1500× magnification, (**B**) 3000× magnification).

Next, we analyzed SEM images of samples obtained using **Methods 1–3**. In the SEM images of composites prepared by **Method 1**, an uneven distribution of **CB[6]** is observed, appearing as conglomerates of molecules on the surface (Figure 7A). This conglomerate formation is likely due to the tendency of **CB[6]** molecules to self-assemble on the **HA** surface under these conditions. When examining the composite in a closer approximation (Figure 7B), one can notice that **CB[6]** on the **HA** surface consists of both conglomerates of **CB[6]** molecules and independent needle-shaped crystals. The formation of conglomerates of **CB[6]** molecules can be stimulated by the process of interaction of **CB[6]** with water, with the formation of aqua complexes of various compositions, which promotes the interaction of crystals with each other, thereby forming such an architecture of **CB[6]** conglomerates. Based on the above, we can conclude that application me **Method 1** does not allow uniform coverage of the **HA** scaffold.

**Figure 8:**
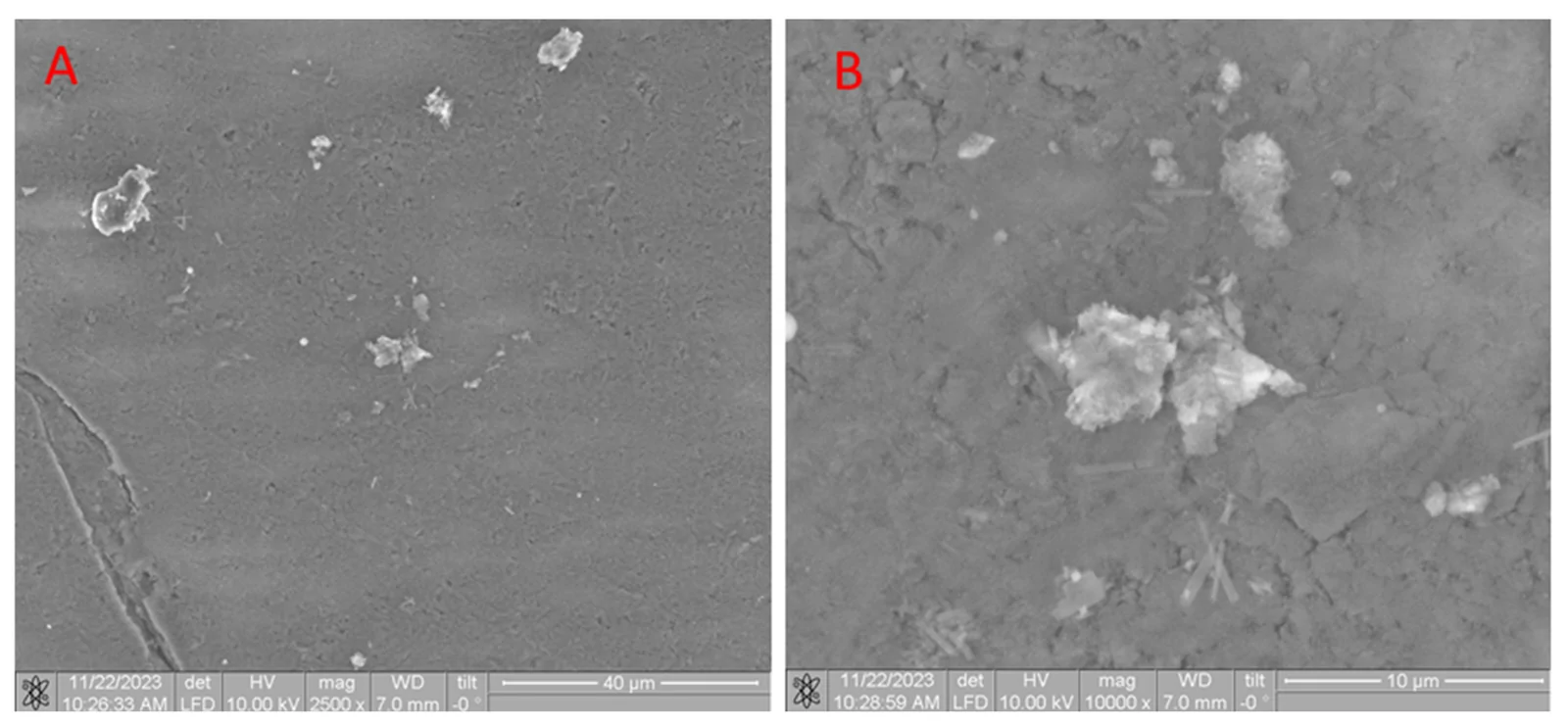
SEM images of samples obtained by **Method 2** ((**A**) 1500× magnification, (**B**) 10,000× magnification). Reprinted from Ref. [26].

**Figure 9:**
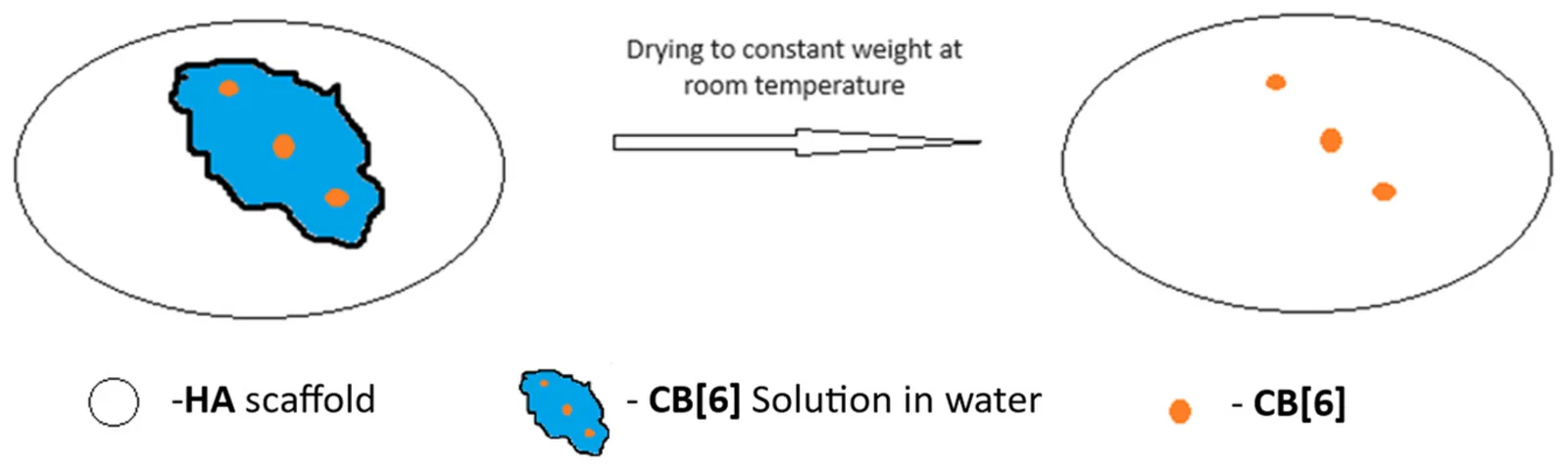
Schematic representation of the formation of conglomerates of **CB[6]** molecules on the surface of **HA** using **Method 2**.

The SEM picture of the samples obtained by **Method 2** on the **HA** surface indicates a comparatively smaller number of **CB[6]** molecules (Figure 8). This statement is confirmed by examining SEM images at different zooms and comparing them with the images of composites obtained using Method 1 (Figure 7A and Figure 8A). Isolated needle-shaped crystals of **CB[6]** are also observed on the **HA** surface. When zooming in 10,000 times, it is clear that the **CB[6]** molecules that are on the surface are formed mainly by conglomerates (Figure 8B). This observed fact is due to the fact that **CB[6]** was applied in dissolved form, and the residual aqueous layer of the solution, which was not removed from the surface due to the wettability effect (Figure 9), contributed to the formation of conglomerates. The **HA** scaffold was saturated with the solution, and most of the **CB[6]** is located inside the composite structure (as evidenced by the IR spectroscopy results shown in Figure 3). In the IR spectra of cleavage samples obtained by **Method 1**, the absorption bands have extremely low intensity, which indicates an insignificant concentration of **CB[6]** on the cleavages of the obtained composites, compared to **Method 2**.

**Figure 10:**
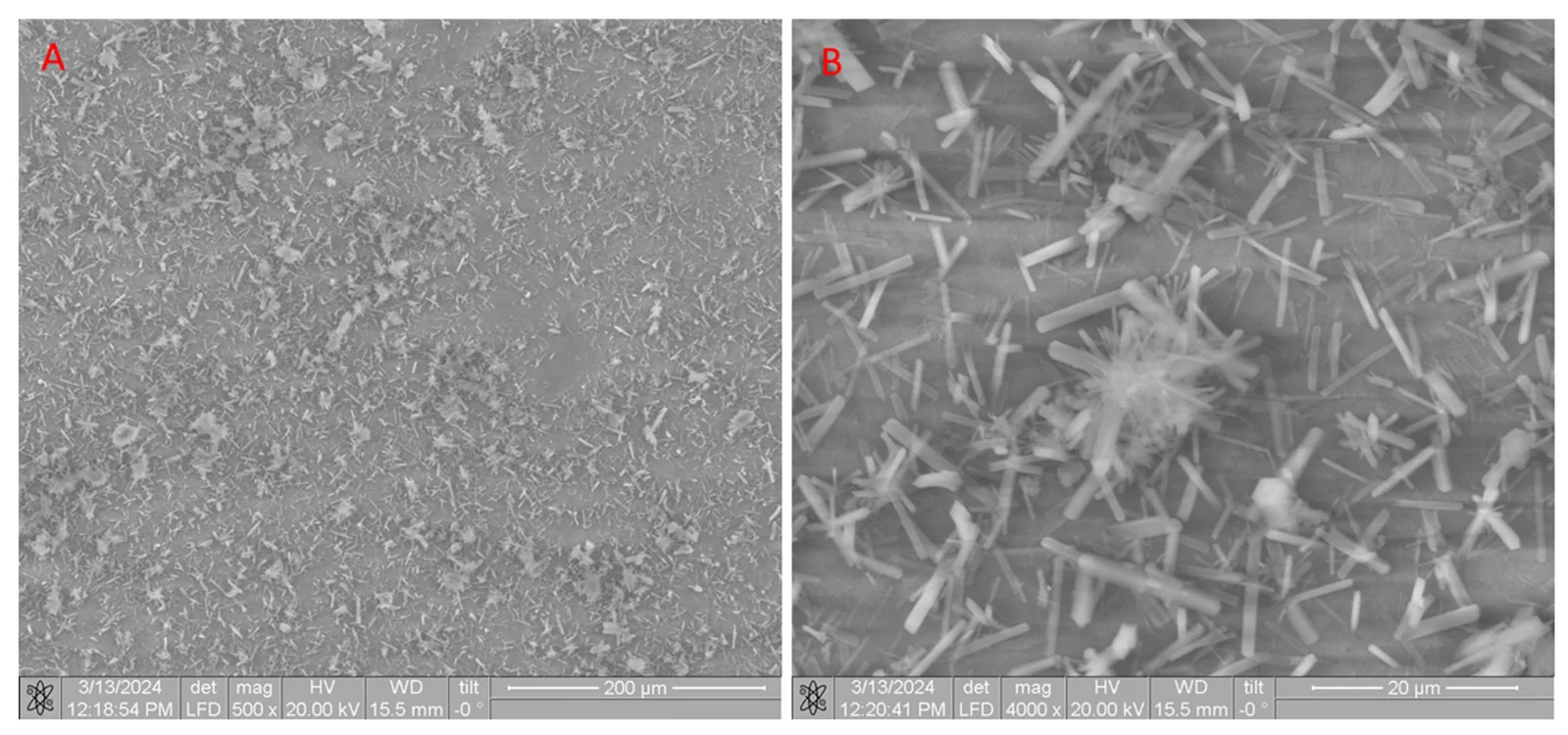
SEM images of samples obtained by **Method 3** ((**A**) 500× magnification, (**B**) 4000× magnification).

When examining SEM images of samples obtained by **Method 3**, a uniform distribution of the **CB[6]** macrocycle on the **HA** surface is observed (Figure 10A). In this case, the formation of needle-shaped crystals is observed over the entire surface of **HA**—the formation of **CB[6]** crystals occurs evenly and simultaneously over the entire surface of the composite. At 4000× zoom (Figure 10B), needle-shaped crystals are seen to cover almost the entire **HA** surface uniformly.

Based on the above, we have proposed a diversification of the scheme for obtaining materials based on **CB[6]** and **HA**. Thus, to uniformly apply **CB[6]** to the surface of materials, various methods can be used, depending on the potential purpose of the resulting materials. In the case of **Method 1**, the surface is covered unevenly; penetration of **CB[6]** into the scaffold does not occur due to low solubility with included water molecules in the cavities of **CB[6]** [33]. In addition, the authors of [34] showed by calculation methods that water molecules in the cucurbit[6]uril cavity are subject to structuring, which is due to their weak interaction with this macromolecule, and the cluster contained in the **CB[6]** cavity has the composition (H_2_O)_6_. This method of modifying the HA surface can be used in cases where a low concentration of **CB[6]** is required on composite surfaces. Using **Method 2**, most of the **CB[6]** is located inside the scaffold, as confirmed by IR spectroscopy and gravimetry. To confirm this, the solution after application (residual solution) was drained and dried to a constant weight and weighed. When studying the residual mass of solutions after completing **Methods 1–3**, it was found that when using **Method 1**, the residual mass was 6 mg, when using **Method 2** it was 5 mg, and when using **Method 3**, it was 3 mg. The advantage of **Method 2** is that when interacting with the internal environment of the body, CB[6] will not be washed out from the surface of **HA**. The peculiarity of **Method 3** is explained by the ability of **CB[6]** to adsorb water, which leads to the penetration of water into the cavity of **CB[6]** and the formation of corresponding aqua complexes [34]. In addition, this method allows for more complete application of **CB[6]** both on the surface and inside the scaffold. When using **Method 3**, the surface is uniformly coated and **CB[6]** also penetrates into the **HA** scaffold, which in turn is the most effective combined option for modifying **HA** scaffolds in the framework of the studies.

**Figure 11:**
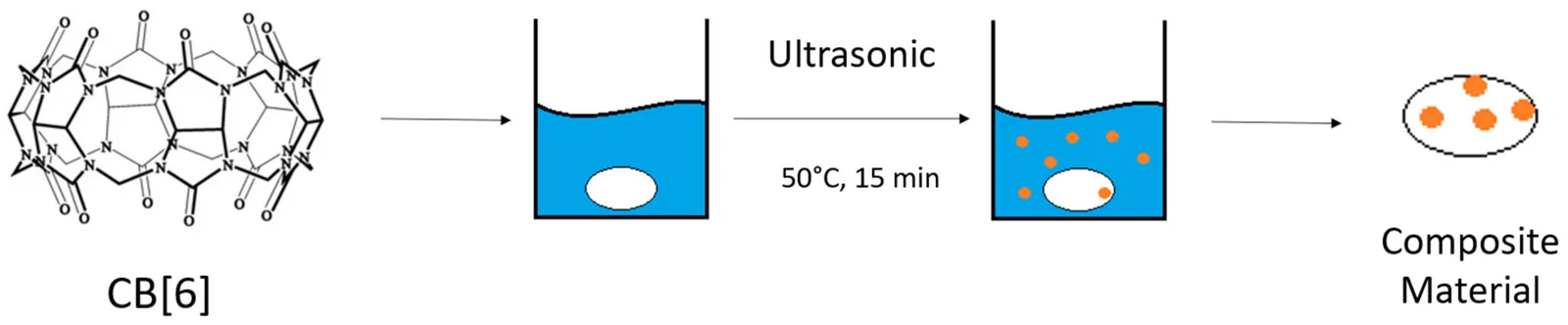
Scheme for producing composites using ultrasonic interaction.

**Figure 12:**
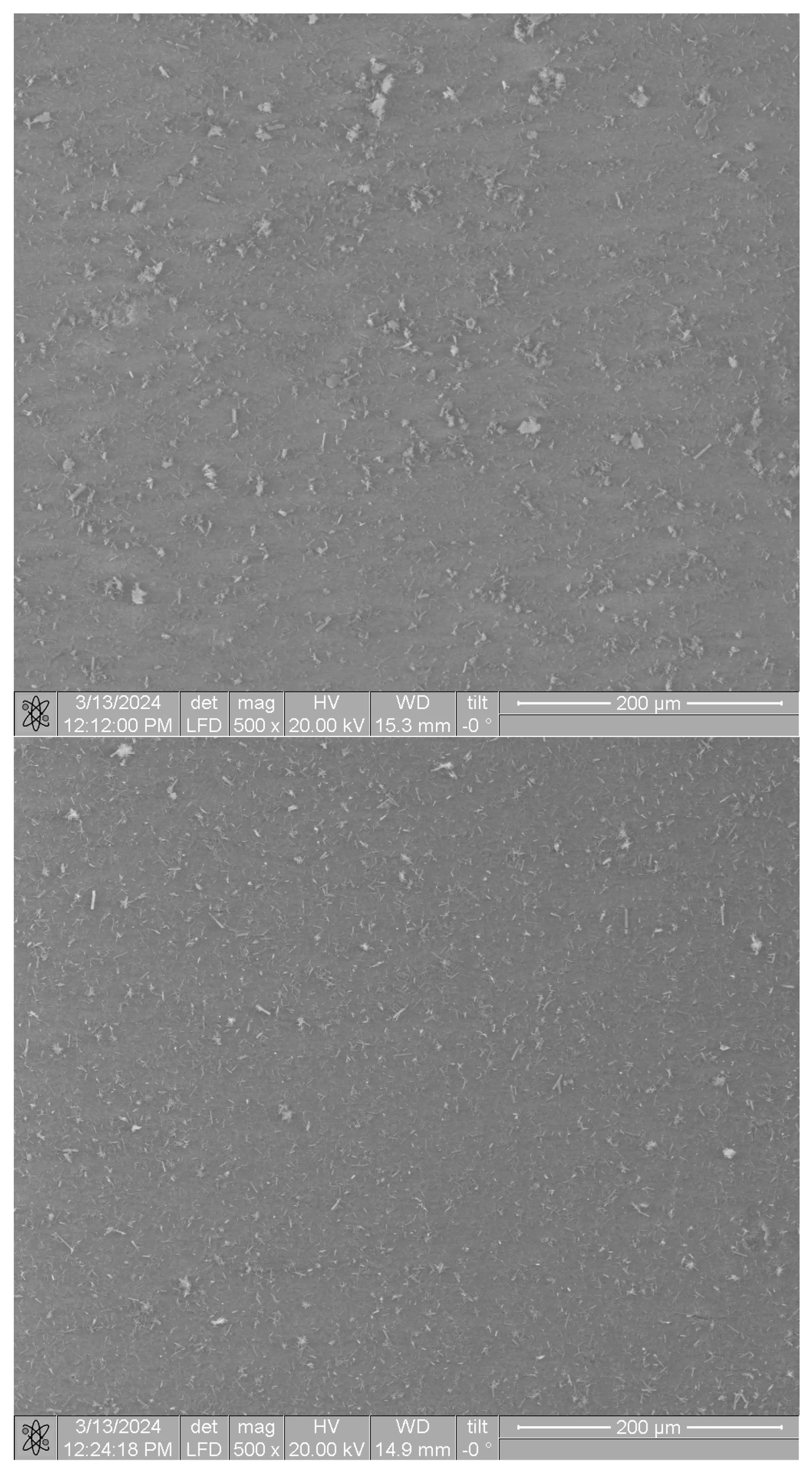
SEM images of composites obtained by **Method 3** (**upper image**) and when composites were obtained by ultrasound interaction (**lower image**)**.**

In development of **Method 3**, the influence of ultrasonic influence on the process of applying **CB[6]** to the **HA** surface was studied. To implement this approach (**Method 4**), the **HA** scaffold was immersed in a solution of 15 mg of **CB[6]** in 15 mL of deionized water located in a Petri dish, then the dish was immersed in an ultrasonic bath with a frequency of 37 kHz. During the experiment, an increase in the temperature of the solution was observed (up to 50 °C for 15 min), and, as a result, cloudiness of the solution occurred (Figure 11). After 20 min, the resulting composite was removed and dried at room temperature to constant weight. In parallel, a similar experiment was carried out, but without external interaction (**Method 3**). The resulting composites were examined using SEM, comparative images of which are shown in Figure 12.

When comparing SEM images of the obtained composites with and without ultrasonic interaction (Figure 12) at the same approximation of 500 times, it is clear that when **HA** is applied using ultrasonic influence on the surface, a more uniform distribution of **CB[6]** over the surface is observed. The number of molecular conglomerates decreases compared to **Method 3**, and at the same time the amount of **HA** surface area free from the macrocycle decreases. This fact indicates that external ultrasonic influence increases the distribution density of **CB[6]** on the **HA** surface.

Moving on to potential applications in the field of personalized medicine, there is the problem of fixation of **CB[n]** with incorporated biological substrates on composite materials, which will be subsequently used in medicine and medical practice. The fundamental issue that needs to be addressed is to ensure that the components are held together by non-covalent interactions. Since the preparation of **CB[n]**-based host–guest complexes as solid products involves mixing hosts and guests in the appropriate stoichiometry, isolation of the complexes in solid form using either lyophilization [35], co-solvent treatment [36], or ball milling [37] is needed. However, the resulting complexes may be inert and not fixed on the surface of the material. Studies of the processes of obtaining biocomposite materials help to choose the necessary and optimal method for obtaining these biomaterials.

## 4. Conclusions

Summarizing the above, it should be noted that, depending on the method of applying **CB[6]** to the surface and inside the **HA** scaffold, it is possible to adjust the composition and structure of the target composite materials. Different application methods in turn increase the variability of the potential applications of composites depending on the practical application. The use of ultrasonic influence increases the surface coverage of **HA** and reduces the number of conglomerates of **CB[6]** molecules, which is apparently due to better wettability of the HA surface and disintegration of **CB[6]** conglomerates under the determined conditions. Methods for applying **CB[6]** to the surface of **HA** have been proposed, which opens the way to functional composite materials based on **CB[6]** and encapsulation products (host–guest complexes).

The proposed methods for applying **CB[6]** to the surface of **HA** impart biodiversity to the latter and provide affinity for the body’s environment when creating new functional composite materials, including the use of supramolecular systems based on the **CB[n]** family.

## Figures and Tables

**Table 1:** Phase composition of HA synthesis products.

Sample	The Inorganic Phase	Parameters of the Electronic Cell, Ǻ	
		a	c
Synthesis Product (**HA**)	Ca_10_(PO_4_)_6_(OH)_2_	9411	6863
JCPDS data, No.9-432	Ca_10_(PO_4_)_6_(OH)_2_	9418	6884

## Data Availability

The data presented in this study are available in this article.
