# Correction: Burkhanbayeva et al. The Role of Methods for Applying Cucurbit[6]uril to Hydroxyapatite for the Morphological Tuning of Its Surface in the Process of Obtaining Composite Materials. *Materials* 2024, *17*, 4995

**DOI:** 10.3390/ma19194204

**Published:** 2026-10-02

**Authors:** Tolkynay Burkhanbayeva, Arthur Ukhov, Dina Assylbekova, Zukhra Mussina, Irina Kurzina, Sandugash Abilkasova, Abdigali Bakibaev, Manar Issabayeva, Rakhmetulla Yerkassov, Zhanat Shaikhova

**Affiliations:** 1 Chemistry Department, Faculty of Natural Sciences, L. N. Gumilyov Eurasian National University, Astana 010000, Kazakhstan; erkass@mail.ru; 2 Chemistry Department, National Research Tomsk State University, Arkady Ivanov St. 49, 634028 Tomsk, Russia; artyryxov1@gmail.com (A.U.); kurzina99@mail.ru (I.K.); bakibaev@mail.ru (A.B.); 3 High School of Chemical Engineering and Biotechnology, M. Auezov South Kazakhstan University, Shymkent 160012, Kazakhstan; asylbekova.dina@inbox.ru; 4 Department of Chemistry, Chemical Technology and Ecology, Almaty Technological University, Almaty 050012, Kazakhstan; muszuhra07@gmail.com (Z.M.); sandugash.abilkasovaa@gmail.com (S.A.); zh.shaikhova1965@gmail.com (Z.S.); 5 Department of Chemistry and Chemical Technology, “Toraighyrov University” NCJSC, Pavlodar 140008, Kazakhstan; isabaeva.manar@mail.ru

In the original publication [1], there was a mistake in the captions of Figures 3 and 8. The corrected captions appear below:

**Figure 3.** X-ray diffraction pattern **CB[6]**. Reprinted from Ref. [26].
**Figure 8.** SEM images of samples obtained by **Method 2** ((**A**) 1500× magnification, (**B**) 10,000× magnification). Reprinted from Ref. [26].

The authors state that the scientific conclusions are unaffected. This correction was approved by the Academic Editor. The original publication has also been updated.
